# 8-Benzoyl-7-hy­droxy-4-methyl-2*H*-1-benzopyran-2-one monohydrate

**DOI:** 10.1107/S1600536810046350

**Published:** 2010-11-13

**Authors:** Shu-Ping Yang, Li-Jun Han, Da-Qi Wang, Xiao-Yun Chen

**Affiliations:** a College of Chemical Engineering, Huaihai Institute of Technology, Lianyungang 222005, People’s Republic of China; b College of Mathematics and Science, Huaihai Institute of Technology, Lianyungang 222005, People’s Republic of China; c College of Chemistry and Chemical Engineering, Liaocheng University, Shandong 252059, People’s Republic of China

## Abstract

In the title compound, C_17_H_12_O_4_·H_2_O, the coumarin ring system is approximately planar with a maximum atomic deviation of 0.011 (2) Å, and is nearly perpendicular to the phenyl ring at a dihedral angle of 86.63 (9)°. In the crystal, mol­ecules are linked by classical O—H⋯O and weak C—H⋯O hydrogen bonds. π–π stacking is also present [centroid–centroid distance = 3.6898 (12) Å].

## Related literature

For the biological activity of coumarins, see: Sharma *et al.* (2005 ▶); Iqbal *et al.* (2009 ▶); Siddiqui *et al.* (2009 ▶); Vyas *et al.* (2009 ▶); Rollinger *et al.* (2004 ▶); Brühlmann *et al.* (2001 ▶). For related structures, see: Yang *et al.* (2006 ▶, 2007 ▶, 2008 ▶).
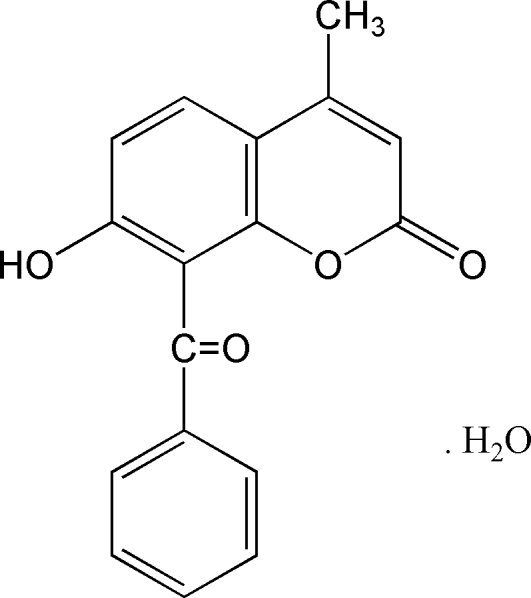


## Experimental

### 

#### Crystal data

C_17_H_12_O_4_·H_2_O
*M* *_r_* = 298.28
Monoclinic, 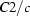

*a* = 14.8912 (15) Å
*b* = 9.6768 (11) Å
*c* = 20.644 (2) Å
β = 104.275 (2)°
*V* = 2882.9 (5) Å^3^
*Z* = 8
Mo *K*α radiation
μ = 0.10 mm^−1^
*T* = 298 K
0.49 × 0.24 × 0.21 mm

#### Data collection

Bruker SMART CCD area-detector diffractometer
Absorption correction: multi-scan (*SADABS*; Sheldrick, 1996 ▶) *T*_min_ = 0.952, *T*_max_ = 0.979
7271 measured reflections
2549 independent reflections
1706 reflections with *I* > 2σ(*I*)
*R*_int_ = 0.036

#### Refinement

*R*[*F* ^2^ > 2σ(*F* ^2^)] = 0.040
*wR*(*F* ^2^) = 0.110
*S* = 1.05
2549 reflections
255 parameters
All H-atom parameters refined
Δρ_max_ = 0.15 e Å^−3^
Δρ_min_ = −0.19 e Å^−3^

### 

Data collection: *SMART* (Siemens, 1996 ▶); cell refinement: *SAINT* (Siemens, 1996 ▶); data reduction: *SAINT*; program(s) used to solve structure: *SHELXTL* (Sheldrick, 2008 ▶); program(s) used to refine structure: *SHELXTL*; molecular graphics: *SHELXTL*; software used to prepare material for publication: *SHELXTL*.

## Supplementary Material

Crystal structure: contains datablocks I, global. DOI: 10.1107/S1600536810046350/xu5087sup1.cif

Structure factors: contains datablocks I. DOI: 10.1107/S1600536810046350/xu5087Isup2.hkl

Additional supplementary materials:  crystallographic information; 3D view; checkCIF report

## Figures and Tables

**Table 1:** Hydrogen-bond geometry (Å, °)

*D*—H⋯*A*	*D*—H	H⋯*A*	*D*⋯*A*	*D*—H⋯*A*
O3—H3⋯O5^i^	0.93 (3)	1.72 (3)	2.650 (2)	177 (3)
O5—H5*A*⋯O2	0.91 (4)	2.00 (4)	2.887 (3)	166 (3)
O5—H5*B*⋯O4^ii^	0.87 (3)	2.03 (3)	2.875 (2)	162 (3)
C17—H17⋯O2^iii^	0.95 (2)	2.54 (2)	3.422 (3)	154.7 (16)

Symmetry codes: (i) 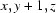; (ii) 
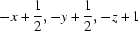
; (iii) 
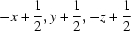
.

**Fig. 1.**
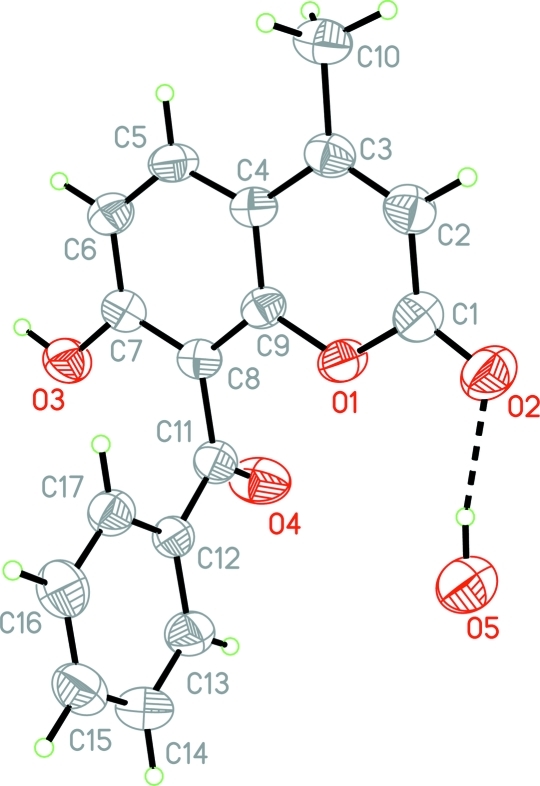
The asymmetric unit of title structure, showing 50% probability displacement ellipsoids for non-H atoms and the atom-numbering scheme, intramolecular O–H···O contact is shown.

**Fig. 2.**
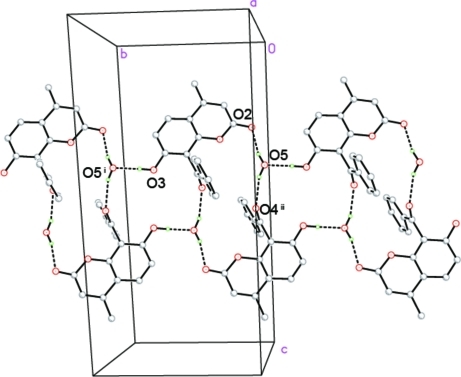
The molecular doubled chain of R56(28)[R44 (20)R44(16)]ring parallel to the b axis. [Symmetry codes: (i) x, 1 + y, z; (ii)1/2 - x, 1/2 - y, 1 - z].

## supplementary crystallographic information

### Comment

Coumarins are very well known for their biological activity, such as antioxidants (Sharma *et al.*, 2005), antiamoebic (Iqbal *et al.*, 2009), anticonvulsant activity (Siddiqui *et al.*, 2009), antimicrobial (Vyas *et al.*, 2009) and inhibitions of acetylcholinesterase and monoamine oxidase (Rollinger *et al.*, 2004; Brühlmann *et al.*, 2001). The crystal structures of some coumarin derivatives (Yang *et al.*, 2006; 2007; 2008) have been decribed. As part of our study of the crystal structures of coumarin derivatives with 7-hydroxy, we report here the crystal structure of 8-Benzoyl-7-hydroxy-4-methyl-2*H*-1-benzopyran-2-one, (I).

In the molecule(I), the asymmetric unit of (I) contains one coumarin molecule and one hydration water molecules, and which are linked together by one O—H···O hydrogen bond (Table 1 and Fig. 1). The coumarin moiety and phenyl ring (two r.m.s deviations 0.0060 Å) are perpendicular to each other with a dihedral angle of 86.59 (5)° between the plane of the atoms O1—O3/C1—C9 and the plane of C12—C17.

In crystal structure of (I), translationally related molecules are linked together by O3—H3···O5^i^ [symmetry code: (i)*x*, 1 + *y*, *z*] hydrogen bond, forming *C*(10) chains parallel to the *b* axis; inversionally related molecular chains are linked together by O—H···O hydrogen bond O5—H5B···O4^ii^ [symmetry codes: (ii)1/2 - *x*, 1/2 - *y*, 1 - *z*], generating doubled chain of *R*_5_^6^(28)[*R*_4_^4^(20)*R*_4_^4^(16)] ring parallel to the *b* axis (Table.1 and Fig. 2). Neighboring doubled chains are linked into three-dimensional crystal structure by π–π interaction *Cg*1···*Cg*1^iii^ [Where *Cg*1 is the centroid of O1/C1—C4/C9, *Cg*1···*Cg*1^iii^ = 3.6898 (12) Å, symmetry code: (iii) -*x*, *y*,1/2 - *z*].

### Experimental

The mixture containing 2.8 g (10 mmol) of dry, powdered 7-benzoxy-4-methylcoumarin and 4.53 g (34 mmol) of anhydrous aluminium chloride was heated at 463 K for 2 h in an oil bath, then 30 ml of dilute (1:7) hydrochloric acid is added and the mixture is heated on a steam bath for 30 min, the crude product was filtered off, washed with water. Colorless crystals of (I) suitable for X-ray structure analysis were obtained by recrystallizing from 95% water-ethanol solution [m.p. 492 K].

### Refinement

All H atom was located in a difference Fourier map and refined freely.

### Crystal data

**Table:**

C_17_H_12_O_4_·H_2_O	*F*(000) = 1248
*M**_r_* = 298.28	*D*_x_ = 1.374 Mg m^−^^3^
Monoclinic, *C*2/*c*	Mo *K*α radiation, λ = 0.71073 Å
Hall symbol: -C 2yc	Cell parameters from 2106 reflections
*a* = 14.8912 (15) Å	θ = 2.6–26.3°
*b* = 9.6768 (11) Å	µ = 0.10 mm^−^^1^
*c* = 20.644 (2) Å	*T* = 298 K
β = 104.275 (2)°	Prism, colorless
*V* = 2882.9 (5) Å^3^	0.49 × 0.24 × 0.21 mm
*Z* = 8	

### Data collection

**Table:**

Bruker SMART CCD area-detector diffractometer	2549 independent reflections
Radiation source: fine-focus sealed tube	1706 reflections with *I* > 2σ(*I*)
graphite	*R*_int_ = 0.036
φ and ω scans	θ_max_ = 25.0°, θ_min_ = 2.0°
Absorption correction: multi-scan (*SADABS*; Sheldrick, 1996)	*h* = −17→14
*T*_min_ = 0.952, *T*_max_ = 0.979	*k* = −11→11
7271 measured reflections	*l* = −24→24

### Refinement

**Table:**

Refinement on *F*^2^	Primary atom site location: structure-invariant direct methods
Least-squares matrix: full	Secondary atom site location: difference Fourier map
*R*[*F*^2^ > 2σ(*F*^2^)] = 0.040	Hydrogen site location: inferred from neighbouring sites
*wR*(*F*^2^) = 0.110	All H-atom parameters refined
*S* = 1.05	*w* = 1/[σ^2^(*F*_o_^2^) + (0.0463*P*)^2^ + 1.197*P*] where *P* = (*F*_o_^2^ + 2*F*_c_^2^)/3
2549 reflections	(Δ/σ)_max_ = 0.001
255 parameters	Δρ_max_ = 0.15 e Å^−^^3^
0 restraints	Δρ_min_ = −0.19 e Å^−^^3^

### Special details

**Table:**

Geometry. All e.s.d.'s (except the e.s.d. in the dihedral angle between two l.s. planes) are estimated using the full covariance matrix. The cell e.s.d.'s are taken into account individually in the estimation of e.s.d.'s in distances, angles and torsion angles; correlations between e.s.d.'s in cell parameters are only used when they are defined by crystal symmetry. An approximate (isotropic) treatment of cell e.s.d.'s is used for estimating e.s.d.'s involving l.s. planes.
Refinement. Refinement of *F*^2^ against ALL reflections. The weighted *R*-factor *wR* and goodness of fit *S* are based on *F*^2^, conventional *R*-factors *R* are based on *F*, with *F* set to zero for negative *F*^2^. The threshold expression of *F*^2^ > σ(*F*^2^) is used only for calculating *R*-factors(gt) *etc*. and is not relevant to the choice of reflections for refinement. *R*-factors based on *F*^2^ are statistically about twice as large as those based on *F*, and *R*- factors based on ALL data will be even larger.

### Fractional atomic coordinates and isotropic or equivalent isotropic displacement parameters (Å^2^)

**Table:**

	*x*	*y*	*z*	*U*_iso_*/*U*_eq_	
O1	0.17438 (9)	0.30443 (14)	0.31197 (6)	0.0429 (4)	
O2	0.13299 (12)	0.09551 (16)	0.27504 (8)	0.0590 (5)	
O3	0.27345 (10)	0.73405 (18)	0.40667 (7)	0.0516 (4)	
H3	0.2699 (19)	0.830 (3)	0.4027 (13)	0.093 (10)*	
O4	0.23351 (10)	0.42720 (19)	0.46551 (7)	0.0621 (5)	
C1	0.12717 (15)	0.2185 (2)	0.26211 (10)	0.0443 (5)	
C2	0.07607 (15)	0.2814 (2)	0.20096 (10)	0.0453 (6)	
H2	0.0427 (14)	0.218 (2)	0.1671 (10)	0.050 (6)*	
C3	0.07320 (13)	0.4192 (2)	0.19106 (9)	0.0397 (5)	
C4	0.12370 (13)	0.5072 (2)	0.24429 (9)	0.0367 (5)	
C5	0.12629 (15)	0.6515 (2)	0.24161 (11)	0.0436 (5)	
H5	0.0918 (13)	0.697 (2)	0.2017 (10)	0.042 (5)*	
C6	0.17489 (14)	0.7290 (2)	0.29421 (11)	0.0438 (5)	
H6	0.1748 (14)	0.829 (2)	0.2898 (10)	0.050 (6)*	
C7	0.22368 (14)	0.6637 (2)	0.35315 (10)	0.0395 (5)	
C8	0.22316 (13)	0.5202 (2)	0.35827 (9)	0.0364 (5)	
C9	0.17343 (13)	0.4459 (2)	0.30380 (9)	0.0359 (5)	
C10	0.01805 (19)	0.4821 (3)	0.12713 (12)	0.0522 (6)	
H10A	−0.0231 (17)	0.414 (3)	0.0985 (12)	0.074 (8)*	
H10B	−0.0219 (17)	0.555 (3)	0.1356 (12)	0.071 (8)*	
H10C	0.0575 (16)	0.525 (3)	0.1039 (12)	0.064 (8)*	
C11	0.27364 (14)	0.4473 (2)	0.42133 (9)	0.0390 (5)	
C12	0.37057 (13)	0.4036 (2)	0.42734 (9)	0.0360 (5)	
H13	0.3868 (14)	0.308 (2)	0.5173 (11)	0.056 (7)*	
C13	0.41872 (16)	0.3316 (2)	0.48382 (11)	0.0484 (6)	
C14	0.51066 (17)	0.2977 (3)	0.49076 (13)	0.0568 (7)	
H14	0.5453 (14)	0.250 (2)	0.5300 (11)	0.057 (6)*	
C15	0.55516 (17)	0.3334 (3)	0.44212 (12)	0.0552 (6)	
H15	0.6208 (17)	0.309 (2)	0.4484 (11)	0.063 (7)*	
C16	0.50801 (16)	0.4022 (3)	0.38535 (12)	0.0523 (6)	
H16	0.5390 (16)	0.430 (2)	0.3503 (12)	0.070 (7)*	
C17	0.41556 (15)	0.4371 (2)	0.37787 (10)	0.0417 (5)	
H17	0.3830 (13)	0.483 (2)	0.3384 (10)	0.046 (6)*	
O5	0.25946 (14)	0.00677 (18)	0.39756 (10)	0.0631 (5)	
H5A	0.220 (2)	0.049 (4)	0.3623 (17)	0.125 (13)*	
H5B	0.258 (2)	0.045 (3)	0.4355 (16)	0.101 (11)*	

### Atomic displacement parameters (Å^2^)

**Table:**

	*U*^11^	*U*^22^	*U*^33^	*U*^12^	*U*^13^	*U*^23^
O1	0.0533 (9)	0.0359 (9)	0.0360 (8)	0.0039 (7)	0.0042 (6)	0.0021 (6)
O2	0.0815 (12)	0.0410 (10)	0.0500 (9)	0.0009 (9)	0.0076 (8)	0.0008 (8)
O3	0.0539 (10)	0.0458 (11)	0.0477 (9)	−0.0017 (8)	−0.0015 (7)	−0.0047 (8)
O4	0.0535 (10)	0.0921 (13)	0.0436 (9)	0.0117 (9)	0.0173 (8)	0.0148 (8)
C1	0.0524 (14)	0.0411 (14)	0.0403 (12)	0.0011 (11)	0.0133 (10)	−0.0015 (10)
C2	0.0499 (14)	0.0491 (15)	0.0357 (12)	−0.0010 (11)	0.0086 (10)	−0.0057 (10)
C3	0.0376 (12)	0.0496 (14)	0.0324 (10)	0.0032 (10)	0.0095 (9)	0.0010 (10)
C4	0.0360 (11)	0.0409 (13)	0.0327 (10)	0.0051 (9)	0.0078 (9)	0.0034 (9)
C5	0.0431 (13)	0.0445 (14)	0.0393 (12)	0.0072 (11)	0.0031 (10)	0.0094 (11)
C6	0.0454 (13)	0.0354 (13)	0.0479 (13)	0.0045 (10)	0.0065 (10)	0.0046 (10)
C7	0.0366 (12)	0.0425 (13)	0.0383 (11)	0.0014 (10)	0.0074 (9)	−0.0020 (10)
C8	0.0351 (11)	0.0401 (13)	0.0339 (10)	0.0035 (9)	0.0080 (9)	0.0017 (9)
C9	0.0373 (11)	0.0340 (12)	0.0374 (11)	0.0049 (9)	0.0108 (9)	0.0027 (9)
C10	0.0494 (15)	0.0626 (18)	0.0403 (13)	0.0049 (14)	0.0029 (12)	0.0046 (12)
C11	0.0427 (12)	0.0404 (12)	0.0329 (11)	−0.0018 (10)	0.0076 (9)	−0.0017 (9)
C12	0.0390 (11)	0.0333 (12)	0.0329 (10)	−0.0020 (9)	0.0039 (9)	−0.0018 (9)
C13	0.0480 (14)	0.0536 (15)	0.0419 (12)	0.0010 (11)	0.0079 (11)	0.0088 (11)
C14	0.0501 (15)	0.0607 (17)	0.0526 (15)	0.0086 (12)	−0.0007 (12)	0.0109 (12)
C15	0.0391 (14)	0.0596 (16)	0.0636 (16)	0.0030 (12)	0.0064 (12)	−0.0049 (13)
C16	0.0473 (14)	0.0588 (16)	0.0534 (14)	−0.0038 (12)	0.0176 (12)	−0.0056 (12)
C17	0.0464 (13)	0.0402 (13)	0.0363 (11)	−0.0016 (10)	0.0065 (10)	0.0000 (10)
O5	0.0828 (13)	0.0548 (12)	0.0488 (11)	0.0045 (9)	0.0106 (10)	−0.0027 (9)

### Geometric parameters (Å, °)

**Table:**

O1—C1	1.374 (2)	C8—C11	1.509 (3)
O1—C9	1.379 (2)	C10—H10A	0.99 (3)
O2—C1	1.218 (2)	C10—H10B	0.96 (3)
O3—C7	1.352 (2)	C10—H10C	0.94 (2)
O3—H3	0.93 (3)	C11—C12	1.479 (3)
O4—C11	1.223 (2)	C12—C17	1.391 (3)
C1—C2	1.438 (3)	C12—C13	1.395 (3)
C2—C3	1.348 (3)	C13—C14	1.381 (3)
C2—H2	0.97 (2)	C13—H13	0.96 (2)
C3—C4	1.446 (3)	C14—C15	1.376 (3)
C3—C10	1.501 (3)	C14—H14	0.96 (2)
C4—C5	1.398 (3)	C15—C16	1.380 (3)
C4—C9	1.400 (3)	C15—H15	0.98 (2)
C5—C6	1.370 (3)	C16—C17	1.389 (3)
C5—H5	0.96 (2)	C16—H16	0.99 (2)
C6—C7	1.404 (3)	C17—H17	0.95 (2)
C6—H6	0.97 (2)	O5—H5A	0.91 (4)
C7—C8	1.393 (3)	O5—H5B	0.87 (3)
C8—C9	1.385 (3)		
			
C1—O1—C9	121.41 (15)	C3—C10—H10A	112.3 (14)
C7—O3—H3	114.9 (17)	C3—C10—H10B	111.4 (15)
O2—C1—O1	115.56 (19)	H10A—C10—H10B	106 (2)
O2—C1—C2	126.8 (2)	C3—C10—H10C	110.6 (14)
O1—C1—C2	117.6 (2)	H10A—C10—H10C	111 (2)
C3—C2—C1	122.8 (2)	H10B—C10—H10C	105 (2)
C3—C2—H2	121.6 (12)	O4—C11—C12	122.57 (18)
C1—C2—H2	115.7 (12)	O4—C11—C8	119.13 (18)
C2—C3—C4	118.45 (18)	C12—C11—C8	118.30 (16)
C2—C3—C10	121.6 (2)	C17—C12—C13	119.26 (19)
C4—C3—C10	119.9 (2)	C17—C12—C11	120.57 (18)
C5—C4—C9	116.35 (19)	C13—C12—C11	120.15 (17)
C5—C4—C3	124.92 (19)	C14—C13—C12	119.8 (2)
C9—C4—C3	118.73 (19)	C14—C13—H13	121.6 (13)
C6—C5—C4	122.0 (2)	C12—C13—H13	118.6 (13)
C6—C5—H5	119.5 (12)	C15—C14—C13	120.5 (2)
C4—C5—H5	118.5 (12)	C15—C14—H14	118.6 (12)
C5—C6—C7	120.0 (2)	C13—C14—H14	120.8 (12)
C5—C6—H6	118.9 (12)	C14—C15—C16	120.3 (2)
C7—C6—H6	121.1 (12)	C14—C15—H15	119.2 (13)
O3—C7—C8	116.86 (18)	C16—C15—H15	120.5 (13)
O3—C7—C6	123.0 (2)	C15—C16—C17	119.7 (2)
C8—C7—C6	120.16 (19)	C15—C16—H16	121.4 (14)
C9—C8—C7	117.90 (18)	C17—C16—H16	118.9 (14)
C9—C8—C11	120.69 (19)	C16—C17—C12	120.4 (2)
C7—C8—C11	121.40 (18)	C16—C17—H17	119.5 (12)
O1—C9—C8	115.39 (16)	C12—C17—H17	120.1 (12)
O1—C9—C4	121.00 (17)	H5A—O5—H5B	112 (3)
C8—C9—C4	123.60 (19)		

### Hydrogen-bond geometry (Å, °)

**Table:**

*D*—H···*A*	*D*—H	H···*A*	*D*···*A*	*D*—H···*A*
O3—H3···O5^i^	0.93 (3)	1.72 (3)	2.650 (2)	177 (3)
O5—H5A···O2	0.91 (4)	2.00 (4)	2.887 (3)	166 (3)
O5—H5B···O4^ii^	0.87 (3)	2.03 (3)	2.875 (2)	162 (3)
C17—H17···O2^iii^	0.95 (2)	2.54 (2)	3.422 (3)	154.7 (16)

Symmetry codes: (i) *x*, *y*+1, *z*; (ii) −*x*+1/2, −*y*+1/2, −*z*+1; (iii) −*x*+1/2, *y*+1/2, −*z*+1/2.
